# Plasmonic Core–Shell–Satellites with Abundant Electromagnetic Hotspots for Highly Sensitive and Reproducible SERS Detection

**DOI:** 10.3390/ijms222212191

**Published:** 2021-11-11

**Authors:** Puran Pandey, Sundar Kunwar, Ki-Hoon Shin, Min-Kyu Seo, Jongwon Yoon, Woong-Ki Hong, Jung-Inn Sohn

**Affiliations:** 1 Division of Physics and Semiconductor Science, Dongguk University-Seoul, Seoul 04620, Korea; ppcpurans@gmail.com (P.P.); kihoonshin@dongguk.edu (K.-H.S.); seominkyuu@gmail.com (M.-K.S.); 2 Los Alamos National Laboratory, Center for Integrated Nanotechnologies (CINT), Los Alamos, NM 87545, USA; sundar@lanl.gov; 3 Jeonju Center, Korea Basic Science Institute, Jeonju 54907, Korea; jwyoon@kbsi.re.kr; 4 Center for Scientific Instrumentation, Korea Basic Science Institute, Daejeon 34133, Korea

**Keywords:** SERS, plasmonic core-shell-satellite, Ag nanoparticles, hotspots, FDTD

## Abstract

In this work, we develop a Ag@Al_2_O_3_@Ag plasmonic core–shell–satellite (PCSS) to achieve highly sensitive and reproducible surface-enhanced Raman spectroscopy (SERS) detection of probe molecules. To fabricate PCSS nanostructures, we employ a simple hierarchical dewetting process of Ag films coupled with an atomic layer deposition (ALD) method for the Al_2_O_3_ shell. Compared to bare Ag nanoparticles, several advantages of fabricating PCSS nanostructures are discovered, including high surface roughness, high density of nanogaps between Ag core and Ag satellites, and nanogaps between adjacent Ag satellites. Finite-difference time-domain (FDTD) simulations of the PCSS nanostructure confirm an enhancement in the electromagnetic field intensity (hotspots) in the nanogap between the Ag core and the satellite generated by the Al_2_O_3_ shell, due to the strong core–satellite plasmonic coupling. The as-prepared PCSS-based SERS substrate demonstrates an enhancement factor (EF) of 1.7 × 10^7^ and relative standard deviation (RSD) of ~7%, endowing our SERS platform with highly sensitive and reproducible detection of R6G molecules. We think that this method provides a simple approach for the fabrication of PCSS by a solid-state technique and a basis for developing a highly SERS-active substrate for practical applications.

## 1. Introduction

The collective oscillation of free electrons in the conduction band of metallic (Au and Ag) nanoparticles (NPs) occurs in interaction with light, namely localized surface plasmon resonance (LSPR) [1]. LSPR effects strongly depend on several parameters of metallic NPs, including their size, shape, spacing, and surrounding medium [2]. Metallic NPs with various shapes, such as nanoplates, nanocages, nanotriangles, nanorods, and nanostars, have been developed to tune the LSPR wavelength in the visible to near-infrared spectrum [3,4,5,6,7]. The tunability of LSPR is crucial to achieving optimal performance in plasmon-mediated applications. The LSPR characteristics of metallic NPs yield strong light absorption and significant amplification of localized electromagnetic coupling at the NPs’ interfaces. Thus, metallic NPs have been utilized as a vital component for potential plasmonic-based applications such as sensing [8], biological imaging [9], energy harvesting [10], surface-enhanced Raman spectroscopy (SERS) [11,12,13,14], and catalysis [15].

SERS is a non-destructive, highly sensitive, and powerful vibrational spectroscopy technique that amplifies the Raman signal of probe molecules and can detect a low concentration of molecules by exploiting the advantages of the LSPR effect on metallic NPs [16,17,18]. Generally, Ag and Au are considered as pivotal materials for highly active SERS substrates due to the intense LSPR and strong electromagnetic (EM) field across the visible region of the electromagnetic spectrum [19,20]. However, bare Ag- or Au-based SERS substrates often face low sensitivity and reproducibility problems mainly because of inadequate control over spatial distribution, interparticle gaps, and size. Therefore, several fabrication methods have been developed to construct highly SERS-active substrates, such as bimetallic alloy, core–shell, core–satellite, and core–shell–satellite nanostructures, etc. [21,22,23,24]. Among these SERS-active nanostructures, the plasmonic core–shell–satellite (PCSS) offers several advantages: (i) protection of the plasmonic core from oxidation, (ii) maintenance of the shape of the core even at high temperatures, (iii) tuning of LSPR properties along with the adjustment of core and satellite size, and (iv) maintenance of small nanogaps between plasmonic core and satellite, producing a strong EM field hotspot [25,26,27,28]. Therefore, PCSS exhibited improved SERS performance due to the strong EM field intensity and abundant built-in EM hotspots. For example, Yang et al. developed a PCSS-based active SERS substrate with a hollow, porous, gold nanoparticle as the core; a protective silica shell; and a gold nanoring satellite for the highly sensitive detection of carcinoembryonic antigen up to 0.1 pg/mL [29]. Similarly, Yang et al. prepared Ag@TiO_2_@Ag PCSS nanostructures, which exhibited ultrasensitive SERS detection of methylene blue molecules (10^−10^ M) and outstanding reproducibility (RSD = 4.02%) due to the plasmon coupling between the Ag core and the Ag satellites and abundant EM hotspots [30]. However, PCSS has been synthesized through a chemical approach, which requires complicated experimental conditions and procedures, involving various chemical reagents. Despite best efforts, the performance of as-produced SERS substrates is greatly affected by residual chemicals. Thus, a simple and highly efficient approach to developing PCSS nanostructures with a high density of intense EM hotspots is urgently required for SERS-based sensors in practical applications.

Here, we proposed and fabricated PCSS (Ag@Al_2_O_3_@Ag) nanohybrid nanostructures as a highly sensitive and reproducible SERS substrate using a simple approach based on a combination of hierarchical film deposition and a dewetting process. The PCSS nanostructures consisted of Ag NPs template as a core, the atomic layer deposition (ALD) of an ultrathin Al_2_O_3_ layer as a shell, and highly dense small Ag NPs on the shell layer as a satellite. The UV–Vis study and the finite-difference time-domain (FDTD) analysis revealed that the LSPR wavelength of PCSS nanostructures is largely shifted toward a longer wavelength compared to bare Ag NPs, and the EM hotspots are also significantly increased due to core-satellite and satellite-satellite plasmonic coupling. The PCSS-based SERS-active substrate exhibited improved SERS sensitivity, with an enhancement factor (EF) of 1.7 × 10^7^ and excellent signal reproducibility with RSD ~7% in the detection of R6G molecules.

## 2. Results and Discussion

**Figure 1:**
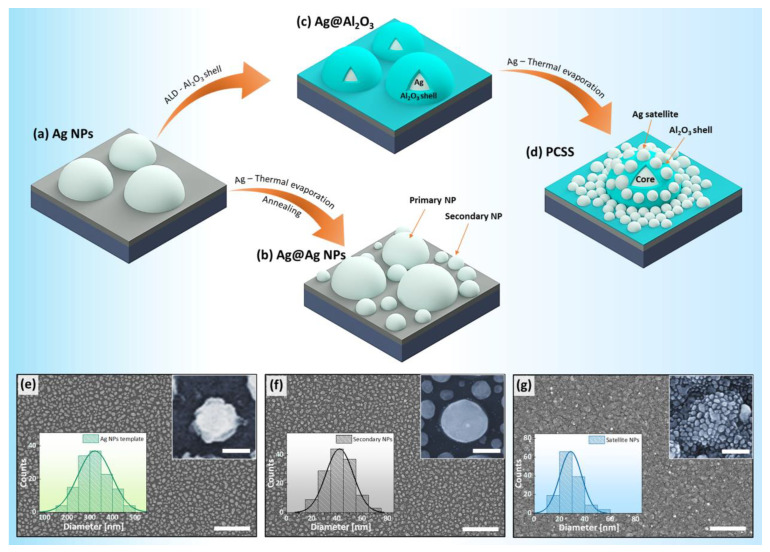
Fabrication procedure for Ag@Ag NPs and plasmonic core-shell-satellite (Ag@Al_2_O_3_@Ag) nanostructures. (**a**) Formation of self-assembled Ag NP template via solid-state dewetting. (**b**) Further coating of the Ag NPs template with thin Ag films followed by low-temperature annealing leading to the formation of Ag@Ag NPs. (**c**) Atomic layer deposition (ALD) of the 3 nm Al_2_O_3_ film on the surface of as-prepared Ag NPs arrays. (**d**) Thermal evaporation of 5 nm Ag thin film on the Ag@Al_2_O_3_ core–shell nanostructures to prepare plasmonic core–shell–satellite (PCSS) nanostructures. (**e**–**g**) SEM images of Ag NP template, Ag@Ag NPs, and PCSS nanostructures. Scale bar: 2 µm. (Insets) Corresponding size distribution histogram and enlarged SEM images of Ag NPs template, secondary Ag NPs, and Ag satellite as labelled. The scale bars in the insets are (**e**) 100 nm, (**f**) 100 nm and (**g**) 130 nm.

Figure 1a–d shows a schematic of the fabrication of PCSS nanostructures and Ag@Ag NPs by combining a film-deposition method and a dewetting process. A detailed synthesis procedure is discussed in the experimental section. Initially, the Ag NPs were formed by thermal dewetting of 15 nm Ag films at 600 °C for 120 s. The surface morphology and thickness of the Ag films are confirmed by the top-view and cross-sectional SEM images in Figure S1. The evolution of Ag NPs from thin Ag films is driven by the combined effect of surface diffusion, Rayleigh instability, and minimization of the surface energy metal substrate system [31,32]. Ag NPs were randomly distributed on a substrate with an areal density of 1.92 × 10^9^/cm^2^ and had a broader size distribution with an average diameter of ~321 nm, as illustrated from the scanning electron microscopy (SEM) image and histogram in Figure 1e. These widely spaced random Ag NPs were utilized as a template for the fabrication of Ag@Ag NPs and PCSS nanostructures. To fabricate Ag@Ag NPs, a 5 nm Ag thin film was coated on the primary Ag NPs template and annealed at low temperature (450 °C for 120 s). As shown in Figure 1b,f, Ag@Ag NPs are highly dense compared to primary Ag NPs. The primary Ag NPs were surrounded by smaller secondary Ag NPs formed during the second step of thermal dewetting. The detailed characterization of the surface morphology based on the atomic force microscopy (AFM) analysis is shown in Figure S2, and larger magnification is presented in Figure S3. The average size of secondary Ag NPs was found to be ~42 nm, while the size of primary Ag NPs was increased compared to the Ag NP template, due to the coalescence of the Ag NPs during the second annealing. It was also observed that the interparticle gaps between Ag@Ag NPs were significantly reduced compared to those of the primary NPs. However, the gap between Ag@Ag NPs was still large due to the accelerated agglomeration and coalescence of Ag, which is an undesired condition for a SERS substrate as it provides a less intense EM hotspot. Thus, in order to prevent the coalescence of Ag film, an ultrathin Al_2_O_3_ film of 3 nm was deposited along the shape of the primary Ag NP template by ALD. The highly dense and small Ag satellites were formed during the subsequent thermal evaporation of a 5 nm thick Ag film on the surface of the Ag@Al_2_O_3_ core–shell structure at room temperature due to the high diffusivity of Ag atoms, causing dewetting. These Ag satellites deposited on the Ag@Al_2_O_3_ can induce significantly reduced interparticle gaps in the PCSS nanostructure. As seen in Figure 1g, the Ag satellites are randomly distributed with much-reduced nanoscale spacing on the surface of core–shell nanostructures. The average size of the Ag satellites was determined to be ~29 nm with a narrow size distribution, as illustrated by the size distribution histogram. Thus, it was deduced that these PCSS nanostructures provide higher surface roughness and abundant nanogaps between the core Ag NPs and the satellite Ag NPs, providing a desirable SERS-active substrate for sensing applications.

**Figure 2:**
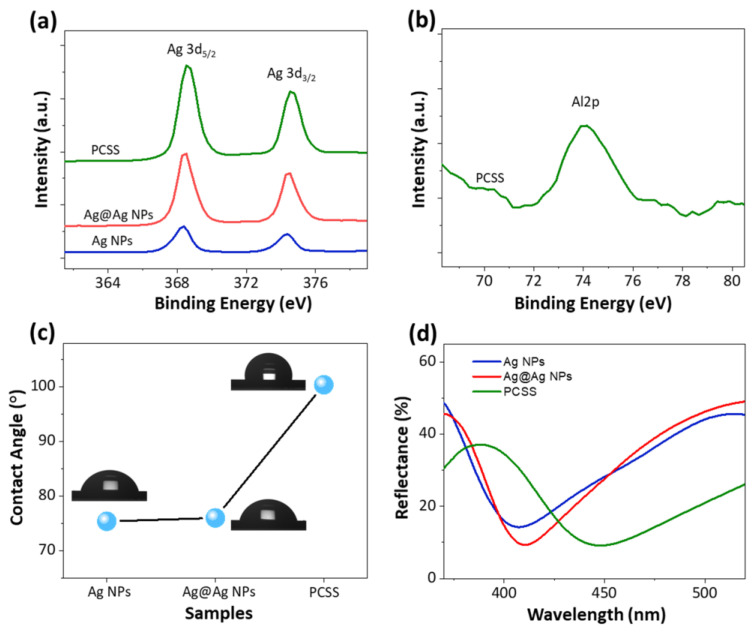
Surface compositional, wettability, and optical characteristics of three different plasmonic nanostructures: Ag NP template, Ag@Ag NP, and PCSS. XPS spectra of (**a**) Ag and (**b**) Al 2p of corresponding nanostructures. (**c**) Water contact angle (WCA) and (**d**) UV–Vis–NIR reflectance spectra of plasmonic nanostructures.

Figure 2a,b show the elemental and chemical analysis of PCSS nanostructures by X-ray photoelectron spectroscopy (XPS) spectra. As shown in Figure 2a, the XPS spectra of Ag 3d exhibit doublet peaks at around 368.3 and 374.3 eV, which are attributed to Ag 3d_5/2_ and Ag 3d_3/2_, respectively, indicating that Ag was mainly in the metallic state [33]. The XPS spectrum of Al 2p in Figure 2b shows a peak at the binding energy of 74.04 eV, which is attributed to the Al_2_O_3_ film [34]. The XPS results confirm the existence of the Ag NPs and the Al_2_O_3_ shell in the PCSS nanostructures. Furthermore, the surface wettability of the Ag NPs template, Ag@Ag NPs, and PCSS nanostructures was studied using a water contact angle test, as shown in Figure 2c. The Ag NP template exhibited a water contact angle of 75.35°. Similarly, the Ag@Ag NPs showed a contact angle of 75.97°, indicating a negligible impact on surface wettability and retention of the hydrophilic nature of Ag NPs. In contrast, the PCSS nanostructures showed a contact angle of 100.39°, indicating an enhancement in the hydrophobic character of the surface. As a result, dispersed probe molecules on the hydrophobic surface of PCSSs can be rapidly concentrated within a tiny spot after the complete evaporation of the solvent, offering superiority in ultra-sensitive detection of probe molecules [35,36].

Figure 2d presents the reflectance spectra of Ag NPs and PCSS nanostructures prepared on a SiO_2_ substrate. The reflectance spectrum of Ag@Al_2_O_3_ nanostructures is presented in Figure S4. It was observed that the reflectance spectrum of Ag NPs shows a plasmonic valley at approximately 407 nm, which can be attributed to the LSPR mode of Ag NPs [37,38]. It is well-known that LSPR wavelength is strongly associated with the size of plasmonic NPs as well as the surrounding medium [25,38,39]. Therefore, for Ag@Ag NPs, the LSPR valley redshifted from 407 to 412 nm due to the slight increase in the size of Ag NPs, arising from the repeated dewetting process. Compared to Ag and Ag@Ag NPs, a large redshift in the LSPR valley to 448 nm was observed for PCSS nanostructures due to the increased refractive index of the surrounding medium in the presence of an Al_2_O_3_ shell [25]. Moreover, although the satellite Ag NPs are much smaller than Ag@Al_2_O_3_, the EM coupling could be largely increased due to the reduced gaps. This could also be another reason for the redshift in the LSPR valley with the PCSS nanostructures. Interestingly, the LSPR valley of PCSSs was found to be broader than the Ag NPs, which is likely due to the wider size distribution of PCSS nanostructures. 

**Figure 3:**
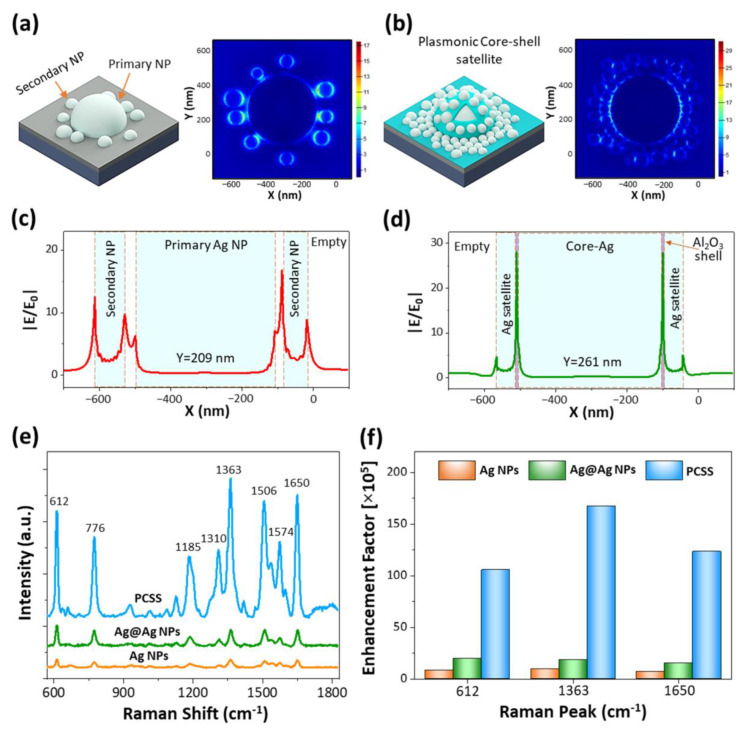
(**a**,**b**) FDTD simulation of Ag@Ag NPs and PCSS nanostructures. The EM-field enhancement was obtained by excitation at 532 nm. (**c**,**d**) Line profile of the EM-field intensity at particular regions of Ag@Ag NPs and PCSS nanostructures. (**e**) SERS spectra of R6G molecules (10^−6^ M) obtained from the Ag NPs template, Ag@Ag NPs, and PCSS nanostructures. (**f**) The plot of the SERS enhancement factor at Raman peaks 612, 1363, and 1650 cm^−1^.

In order to probe the EM field intensity and spatial distribution of the Ag@Ag NPs and PCSS nanostructures, we numerically simulated typical nanostructures using the FDTD method. The simulation model was constructed by acquiring the geometric parameters of typical nanostructures from SEM images. Detailed explanations of the simulation model and parameters used are given in the experimental section and in Figure S5. Figure 3a,b shows the typical simulation model and EM field maps of Ag@Ag NPs and PCSS nanostructures under 532 nm excitation. As seen in the FDTD simulation of Ag@Ag NPs, several EM hotspots formed at the nanogap between the primary and secondary Ag NPs due to the plasmonic coupling. In particular, intensive EM field hotspots were generated at narrow nanogaps, as confirmed by the line profile of the EM field in Figure 3c. More interestingly, the PCSS nanostructures demonstrate the high density and intense EM field hotspots due to multiple plasmonic coupling effects, including Ag core–Ag satellite coupling within the 3 nm nanogap formed by the Al_2_O_3_ shell and Ag satellite–satellite coupling within a narrow nanogap. The line profile of the EM field intensity distribution at specific locations of PCSSs in Figure 3d shows a stronger EM field enhancement in the 3 nm thick Al_2_O_3_ shell region serving as the nanogap spacer compared to Ag@Ag NPs. Moreover, the enhancement of the SERS signal is approximately proportional to the fourth power of the local EM field intensity (i.e., EF = |E|^4^/|E_0_|^4^) [40], suggesting that the PCSS nanostructures provide the most abundant and intense hotspots suitable for highly sensitive and reproducible detection of probe molecules.

To assess the capability of SERS detection of as-prepared substrates, R6G was used as the probe molecule. Figure 3e shows the comparative SERS enhancement effect of three different SERS substrates, i.e., Ag NP template, Ag@Ag NPs, and PCSS nanostructures with the same concentration of R6G (10^−6^ M). For all SERS-active substrates, the prominent characteristic Raman peaks of R6G were observed at 612, 776, 1185, 1310, 1363, 1506, and 1650 cm^−1^ under excitation at 532 nm, which are in agreement with the previously reported values [41,42]. The Raman peaks at 612, 776, 1185 and 1310 cm^−1^ correspond to C–C–C in-plane ring vibration, out-of-plane bending motion, C-H plane bending, and C–O–C stretching, respectively. Other peaks observed at 1363, 1506, and 1650 cm^−1^ are attributed to the C–C stretching of the aromatic ring. As observed in Figure 3e, the intensity of the Raman peaks drastically varied depending on the SERS substrate. In particular, the SERS signal was successively enhanced with PCSS nanostructures compared to the primary Ag NP template and Ag@Ag NPs substrate with constant R6G concentration. The SERS signal enhancement from PCSSs is in agreement with the FDTD simulation results, showing more intense hotspots generated by the Al_2_O_3_ shell, providing a 3 nm spacing between the Ag core and satellite. Another important factor influencing the enhancement of SERS performance is the increase in the hydrophobic nature of the PCSS nanostructures by rapidly concentrating the R6G molecules within a smaller area of the substrate [43,44]. In order to quantitatively compare the performance of SERS for three different samples, the enhancement factor (EF) was estimated according to the equation EF = (I_sers_ / C_sers_) / (I_ref_ / C_ref_), where I_sers_ and I_ref_ are the intensities of the Raman peaks of R6G molecules adsorbed on SERS and the reference substrate (SiO_2_/Si), respectively; C_sers_ (10^−6^ M) and C_ref_ (10^−2^ M) are the concentrations of R6G molecules on SERS and the reference substrate, respectively. The calculated SERS EFs for three different SERS substrates at strong Raman peaks 612, 1363, and 1650 cm^−1^ are plotted in Figure 3f and summarized in Table S1. The SERS EF of the PCSS nanostructures for the Raman peak 1364 cm^−1^ is 1.7 × 10^7^, which is almost 17 and 9 times higher than those of the Ag NP template and Ag@Ag NPs, respectively.

**Figure 4:**
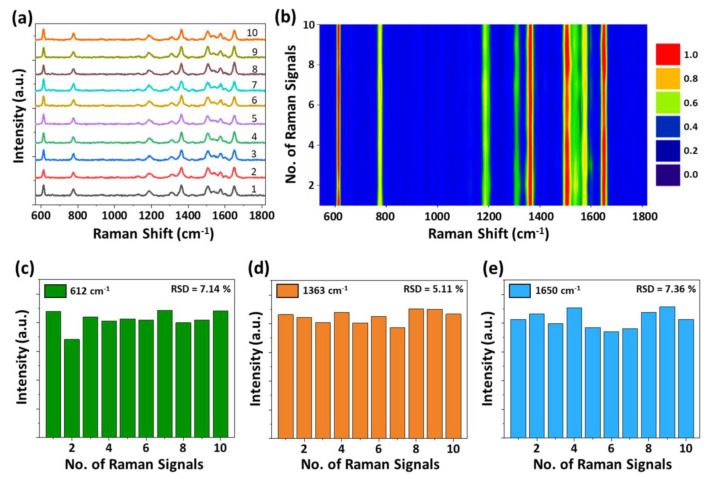
Reproducibility of SERS signals on a PCSS nanostructure-based SERS substrate. (**a**) SERS spectra of R6G molecules recorded from 10 different spots on the SERS substrate. (**b**) The corresponding SERS contour from a line mapping of 10 spots. (**c**–**e**) SERS intensity distribution of the 612, 1363, and 1650 cm^−1^ Raman peaks.

The reproducibility of SERS signals is another important factor for evaluating SERS-active substrates for practical applications. The SERS spectra of R6G molecules were randomly measured at 10 different locations on the PCSS SERS substrate to examine reproducibility, as shown in Figure 4a. The corresponding color contour plot of the SERS spectra is presented in Figure 4b. All the SERS spectra obtained from different locations showed similar peak positions and intensities, signifying impressive uniformity of the SERS substrates. The corresponding relative standard deviation (RSD) values of the major Raman peaks at 612, 1363, and 1650 cm^−1^ were calculated from the Raman intensities acquired at 10 spots of the SERS substrate as shown in Figure 4c–e. The RSD values were found to be 7.1%, 5.1%, and 7.3 % for peaks 612, 1363, and 1650 cm^−1^, respectively, confirming the excellent reproducibility of the PCSS-based SERS substrate. 

## 3. Materials and Methods

### 3.1. Fabrication of Ag NP Templates and Ag@Ag NPs

First, the SiO_2_/Si substrates (purchased from Sehyoung wafertech, Seoul, Korea) were cleaned with acetone, isopropyl alcohol (IPA), and DI water in an ultrasonic bath for 10 min each and finally dried by compressed nitrogen (N_2_) gas. Then, we deposited 15 nm Ag films on the cleaned SiO_2_/Si substrates via thermal evaporation. Subsequently, the Ag films were annealed in a rapid thermal annealing (RTA) chamber at 600 °C for 120 s to form Ag NPs. As-formed Ag NPs were used as a template for further fabrication of different plasmonic nanostructures. To fabricate Ag@Ag NPs, we deposited 5 nm Ag films on the Ag NP template via thermal evaporation, and subsequently annealed at 450 °C for 120 s. In the second step, annealing was performed at a lower temperature to prevent the agglomeration of secondary NPs with the template NPs.

### 3.2. Fabrication of Plasmonic Core–Shell Satellites

Plasmonic core–shell satellite NPs were prepared on the Ag NPs template by combining two processes: (i) Al_2_O_3_ shell layer deposition and (ii) Ag film deposition. In particular, the Al_2_O_3_ shell with a 3 nm thickness was coated on the Ag NPs template using an ALD method. The ALD deposition of Al_2_O_3_ was carried out using the precursors trimethylaluminum (Al(CH_3_)_3_, TMA) and ozone (O_3_) at a temperature of 220 °C and pressure of 0.1 Torr. Subsequently, a 5 nm thick Ag film was coated on the core–shell Ag@Al_2_O_3_ nanostructures by thermal evaporation at room temperature. The thin Ag films deposited on Ag@Al_2_O_3_ nanostructures were dewetted to form Ag satellites due to the high diffusivity of Ag atoms, which are termed plasmonic core–shell–satellite nanostructures.

### 3.3. Sample Characterization and SERS Measurement

The surface morphology of the nanostructures was characterized by scanning electron microscope (SEM) and atomic force microscope (AFM). The size of the nanostructures was acquired from SEM images using ImageJ software. The surface chemical states of the fabricated nanostructures were examined by X-ray photoelectron spectroscopy (XPS). We carried out water contact angle (WCA) measurements to assess the surface wettability of plasmonic nanostructures at room temperature using a CA measurement instrument (JCA-1). The volume of the drop was 3 µL, and the WCA value was measured three times on the same sample surface to ensure statistical precision. The optical properties of the nanostructures were characterized by a UV–Vis–NIR reflectance spectrometer. To evaluate the SERS performance, the prepared SERS substrates were immersed in a 10^−6^ M R6G solution (prepared in ethanol) for 2 h and naturally dried at room temperature. The SERS signals of R6G molecules were examined using confocal Raman spectroscopy (HEDA, NOST) at room temperature. A 532 nm laser (power of 0.1 mW) with a 100× objective lens (numerical aperture = 0.80) and an acquisition time of 10 seconds was used for all SERS measurements.

### 3.4. FDTD Simulation

The FDTD method (Lumerical Solutions Inc., Vancouver, BC, Canada) was used to calculate the distribution of the electromagnetic field intensity around the nanostructures. In the simulation model of Ag@Ag NPs, the diameter of the primary Ag NPs was 400 nm, while the size of the secondary NPs varied between 40 and 75 nm. The spacing of Ag NPs was estimated to be between 7 and 10 nm based on the SEM results. For modeling PCSS nanostructures, the diameter of the Ag core and the Ag satellite was 400 and 30–60 nm, respectively, and the thickness of Al_2_O_3_ was set to 3 nm according to the experimental measurements. The surrounding medium of the nanostructures was set to air. A perfectly matched layer (PML) was used in the z-boundaries and the periodic boundary condition was selected for the x and y directions. A 0.3 nm mesh size and a 532 nm plane wave were chosen for simulations.

## 4. Conclusions

We successfully demonstrated the creation of a highly sensitive and reproducible SERS substrate with PCSS nanostructures consisting of a Ag NP core, an Al_2_O_3_ shell, and small Ag NP satellites, fabricated with a simple hierarchical dewetting process coupled with an ALD method. We demonstrated that the PCSS nanostructures can lead to the formation of several EM hotspots between the Ag core and the Ag satellites within the Al_2_O_3_ gap regions, as well as nanogaps between the Ag–Ag satellite. Compared to Ag NPs, PCSS nanostructures exhibit abundant strong EM field hotspots due to the strong coupling between the Ag core and the Ag satellite in the Al_2_O_3_ shell region, as supported by the FDTD simulation of the core–shell–satellite assembly. The sensitivity and reproducibility of PCSS nanostructures for SERS applications were evaluated by R6G dye molecules. The SERS enhancement factor of the PCSS-based substrate reached up to 1.7 × 10^7^, which is a considerable improvement compared to bare Ag NPs. In addition, the RSD values of the three Raman peaks of R6G were ~7%, demonstrating the excellent reproducibility of the PCSS-based SERS substrate.

## Data Availability

Data are contained within the article or supplementary material.

## Supplementary Materials

The following are available online at https://www.mdpi.com/article/10.3390/ijms222212191/s1, Figure S1. (a) SEM image of Ag films (15 nm) deposited on SiO_2_/Si substrate via thermal evaporation. (b) Corresponding cross-sectional SEM image to demonstrate the actual thickness of Ag films; Figure S2. Analysis of the surface morphology of Ag@Ag NPs and PCSS nanostructures using AFM data; Figure S3. SEM images of (a) Ag@Ag NPs, and (b) PCSS nanostructures; Figure S4. Reflectance spectrum of core–shell Ag@Al_2_O_3_ nanostructures; Figure S5. Schematic representation of the FDTD simulation model used for the calculation of the E-field enhancement. Table S1. Summary of the SERS enhancement factor (EF) for Ag NPs, Ag@Ag NPs, and PCSS nanostructures at Raman peaks 612, 1363, and 1650 cm^−1^.
