# Supplementary material for: Plasmonic Core–Shell–Satellites with Abundant Electromagnetic Hotspots for Highly Sensitive and Reproducible SERS Detection
Source: Int J Mol Sci. 2021 Nov 11;22(22):12191. doi: 10.3390/ijms222212191 (PMC8620478; doi:10.3390/ijms222212191)
Supplement: Supplementary file 1 [file ijms-22-12191-s001.zip › ijms-1438223-supplementary.pdf]

Supplementary Material

Plasmonic Core-Shell-Satellites with Abundant Electromagnetic Hotspots for Highly Sensitive and Reproducible SERS Detection

Puran Pandey [1], Sundar Kunwar [2], Ki Hoon Shin [1], Min-Kyu Seo [1], Jongwon Yoon [3], Woong-Ki Hong [4],* and Jung Inn Sohn [1,*]

[1] Division of Physics and Semiconductor Science, Dongguk University-Seoul, Seoul 04620 Republic of Korea; ppcpurans@gmail.com; kihoonshin@dongguk.edu; seominkyuu@gmail.com

[2] Center for Integrated Nanotechnologies (CINT), Los Alamos National Laboratory, Los Alamos, New Mexico 87545, USA; sundar@lanl.gov

[3] Jeonju Center, Korea Basic Science Institute, Jeonju 54907, Republic of Korea; jwyoon@kbsi.re.kr

[4] Center for Scientific Instrumentation, Korea Basic Science Institute, Daejeon 34133, Republic of Korea

* Correspondence: junginn.sohn@dongguk.edu; wkh27@kbsi.re.kr

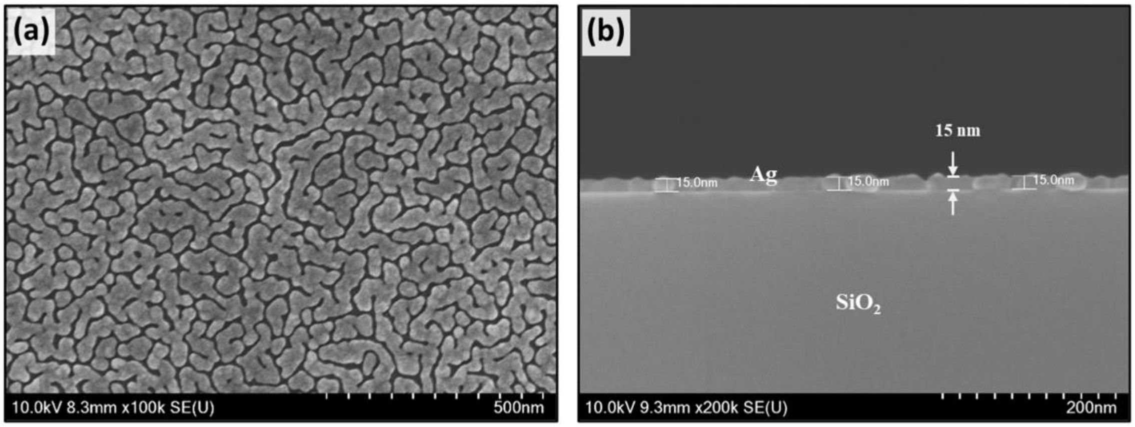

Figure S1. (a) SEM image of Ag films (15 nm) deposited on SiO_2/Si substrate via thermal evaporation. The morphology of the Ag films is discontinuous porous structure. (b) Corresponding cross-sectional SEM image to demonstrate the actual thickness of Ag films.

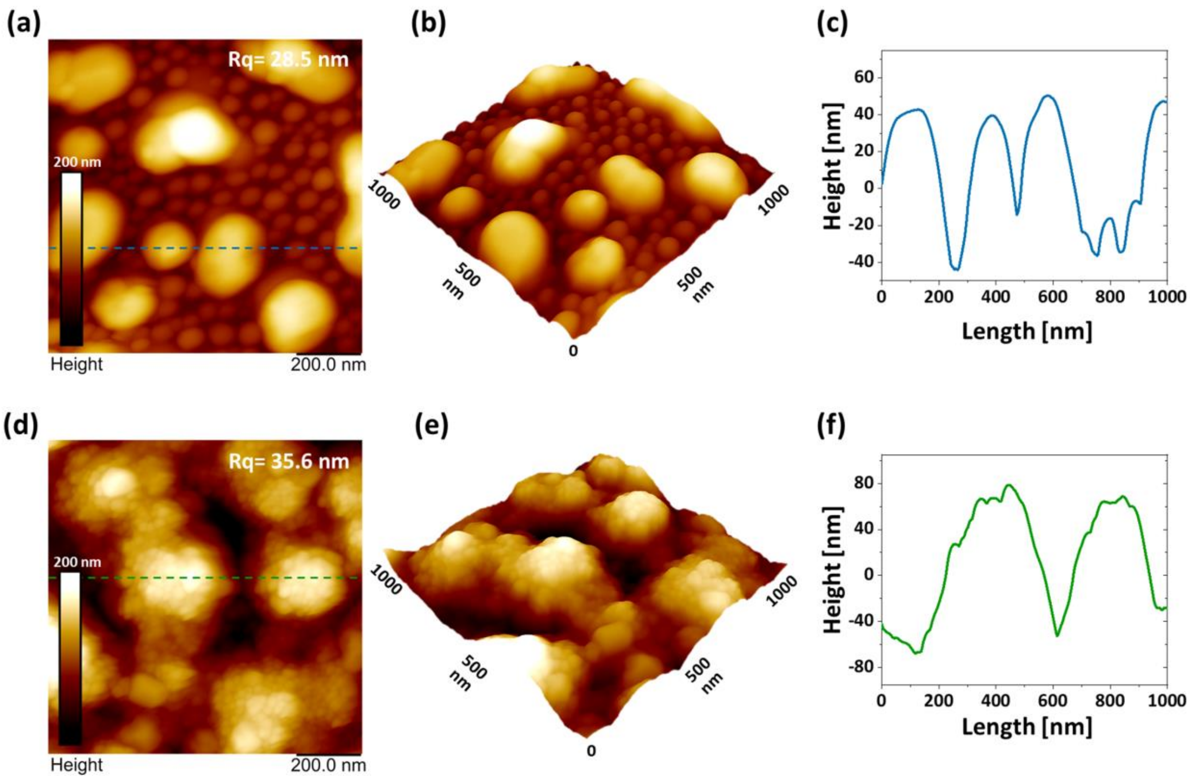

Figure S2. Analysis of the surface morphology of Ag@Ag NPs and PCSS nanostructures using AFM data. **(a)** and **(d)** AFM top-views of Ag@Ag NPs and PCSS nanostructures, respectively. **(b)** and **(d)** Corresponding AFM side views. **(c)** and **(f)** Cross-sectional line profiles to reveal height information. The dashed lines in **(a)** and **(d)** denote the specific location of the line profile. The surface roughness of the PCSS nanostructures is observed to be 35.6 nm, which is much higher than that of the Ag@Ag NPs (27.0 nm).

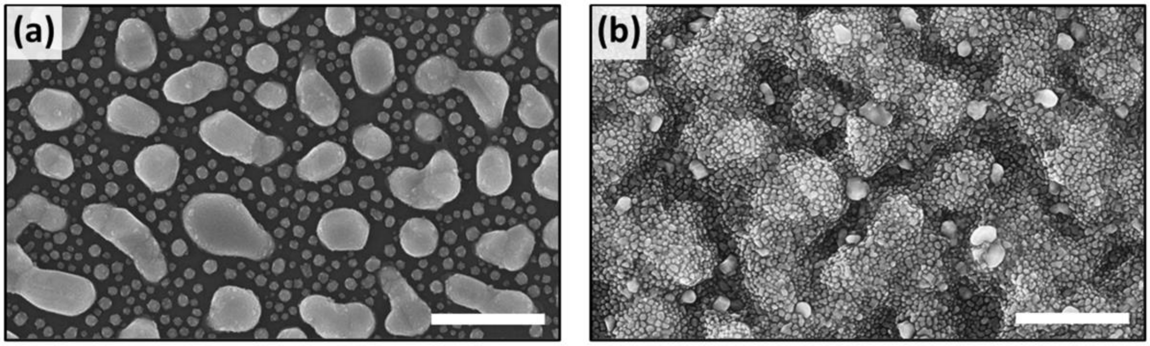

Figure S3. SEM images of (a) Ag@Ag NPs, and (b) PCSS nanostructures. Scale bar: 500 nm.

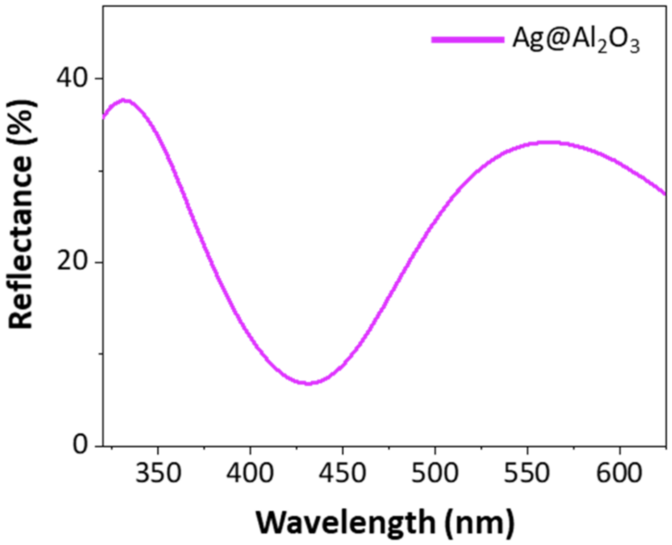

Figure S4. Reflectance spectrum of core-shell Ag@Al_2O_3 nanostructures.

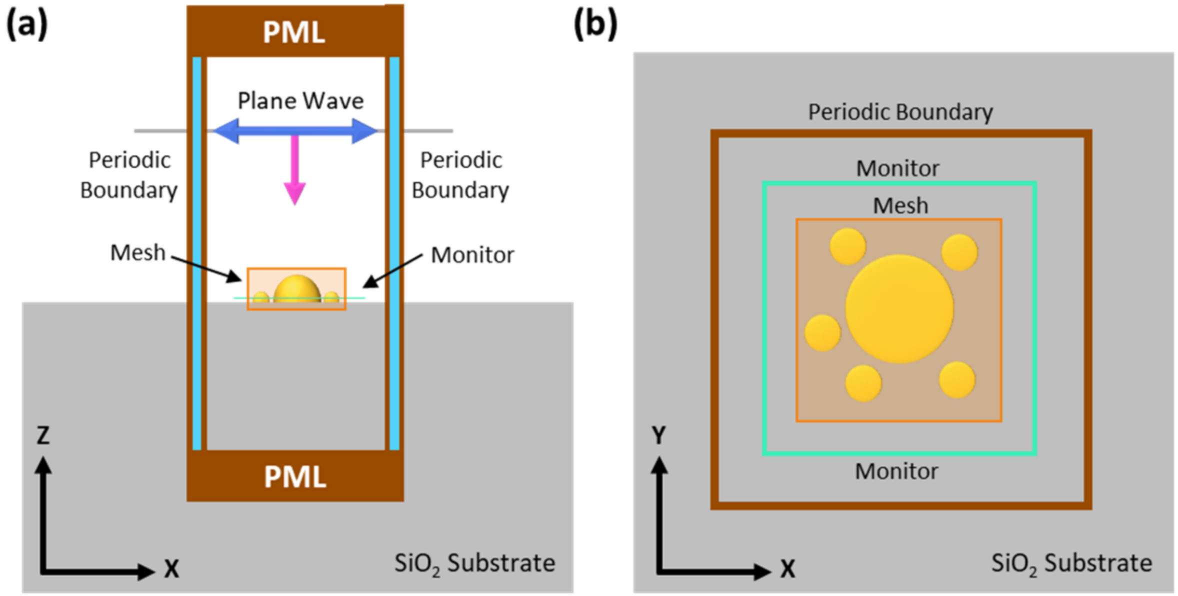

Figure S5. Schematic representation of the FDTD simulation model used for the calculation of the EM field enhancement. **(a)** X-Z view and **(b)** X-Y view of the simulation model.

Tables S1. Summary of the SERS enhancement factor (EF) for Ag NPs, Ag@Ag NPs, and PCSS nanostructures at Raman peaks 612, 1363, and 1650 cm^{-1}.

S. N.	SERS Substrate	Raman Peaks	Enhancement Factor (EF)
1	Ag NPs	612 cm^{-1}	8.3×10^5
		1363 cm^{-1}	9.4×10^5
		1650 cm^{-1}	7.3×10^5
2	Ag@Ag NPs	612 cm^{-1}	1.9×10^6
		1363 cm^{-1}	1.8×10^6
		1650 cm^{-1}	1.5×10^6
3	PCSS nanostructure	612 cm^{-1}	1.1×10^7
		1363 cm^{-1}	1.7×10^7
		1650 cm^{-1}	1.2×10^7
